# Sensitivity of candling as routine method for the detection and recovery of ascaridoids in commercial fish fillets

**DOI:** 10.1038/s41598-022-05235-6

**Published:** 2022-01-25

**Authors:** E. Mercken, I. Van Damme, B. Šoba, S. Vangeenberghe, A. Serradell, T. De Sterck, J. P. L. Lumain, S. Gabriël

**Affiliations:** 1 Laboratory of Foodborne Parasitic Zoonoses, Faculty of Veterinary Medicine, Ghent University, Salisburylaan 133, Merelbeke, Belgium; 2 Institute of Microbiology and Immunology, Faculty of Medicine, University of Ljubljana, Ljubljana, Slovenia

**Keywords:** Infectious-disease diagnostics, Parasitology

## Abstract

Ascaridoids are one of the main parasitic hazards in commercial fish. Candling is the current industrial screening method whereby visible ascaridoid larvae are detected on a light table and manually removed. The aim of this study was to assess the sensitivity (Se) and negative predictive value (NPV) of this method. To make targeted recommendations to the fish industry, the Se was calculated per fish part, larval genus, and fish species. All fish parts (n = 615) were first candled, and larvae were collected, followed by enzymatic digestion to recover the remaining larvae. A fish part was considered positive if at least one larva was detected using candling and/or enzymatic digestion, with both methods combined as reference standard. The overall Se of candling was 31% (95% CI 23–41%) and NPV was 87% (95% CI 85–90%). The Se increased with higher numbers of larvae/100 g infected muscle. A low NPV was found for the belly flaps, therefore we either advise the removal or proper freezing of this part. Lastly, the Se and larval recovery was the highest for the darker and larger *Pseudoterranova* spp. larvae. Due to the low overall efficacy of candling, further assessment of its cost–benefit and impact on consumers’ health risk should be conducted.

## Introduction

One of the important biological hazards in seafood is the third stage (L3) larva of the genera *Anisakis*, *Pseudoterranova,* and *Contracaecum*^1^, and to a lesser extend *Hysterothylacium*^2,3^. Humans can become an accidental host after the ingestion of a viable L3 larva inside raw or undercooked infected fish. Symptoms range from gastro-intestinal complaints to allergic reactions against the (heat-resistant) allergens^4,5^. The lifecycle of these ascaridoids includes marine mammals as final host, crustaceans as intermediate host and fish as paratenic host. In fish, third stage larvae can migrate from the viscera to the muscles, both ante-mortem and post-mortem^6^. Muscle-invading larvae mostly coil up in the belly flaps but can also migrate further away from the abdominal cavity^7–10^. Infected fish lose value or can no longer be sold in the event of a clearly severe infection. In addition, consumers are becoming more concerned about food safety and food quality and are therefore reluctant to buy fishery products once they have noticed a (viable) larva^11,12^. This phenomenon creates economic losses as well as reputation damage for the industry^13^.

Control of this zoonosis focusses on the prevention of post-mortem migration (gutting and immediately storing the fish on ice after the catch), inactivation (freezing or heating), and/or removal of the ascaridoids. European regulation (EC) No 853/2004 states that all fish destined for raw/almost raw consumption must be frozen at a temperature of minimum − 20 °C for at least 24 h and all fish must undergo visual examination to detect and remove all visible parasites^14^. An industrial screening method applied for this is candling, a fast inspection method in which fish fillets are checked on a light table and larvae are manually removed when visible. Factors such as the skills of the examiner; the colour and thickness of the fillets; and the location, size and colour of the larva can play a role in the accuracy of detecting ascaridoids using candling^15,16^. While the sensitivity of candling is commonly accepted as low, in fact only a few studies provide more general data regarding the performance of candling, and indeed indicate a very low efficacy to detect the majority of the larvae^16,17^. The study of Levsen et al.^16^ was limited to mackerel, blue whiting, and herring with only *Anisakis* spp. being detected (however no microscopic or molecular species identification was performed). Though prevalence was variable for the three fish species, a similar recovery of 7–10% of the larvae was reported for the whole fish fillet. Petrie et al.^17^ analysed only four different fish species and reported a higher candling efficacy (50%) for cod and monkfish compared to herring and mackerel.

Over the years, researchers have looked into the development of more accurate, fast, industrial applicable screening methods, but none were able to replace candling so far^18^. Although highly accurate methods, such as enzymatic digestion and the ultra–violet (UV) press method, are available and are commonly used in research^19,20^, the complete destruction of the fish renders these methods unusable at a large scale in the food industry. Despite the high labour cost, candling is therefore still the most routinely used method for the detection of ascaridoids in commercial fish fillets and a more in-depth assessment of its performance is needed.

The aim of this study was to conduct an in-depth evaluation of the diagnostic accuracy of candling. The performance of candling was assessed for the specific fish parts (anterior, belly flaps, medial, and posterior part). Moreover, performance assessments were made related to the number of larvae and larvae species. As a secondary aim, we evaluated candling performance on fish species level.

With the obtained information we aimed to determine where the application of the candling technique could still be beneficial, thereby providing recommendations to the fish industry for a more targeted use.

## Materials and methods

### Sample collection and processing

Dead whole fish (N = 205) were collected from a Belgian whole-sale company during a year-round cross-sectional study, between May 2018 and May 2019. Fish were selected from the available fish species. More details of the sampling and sampling areas can be found in Mercken et al.^8^.

**Figure 1:**
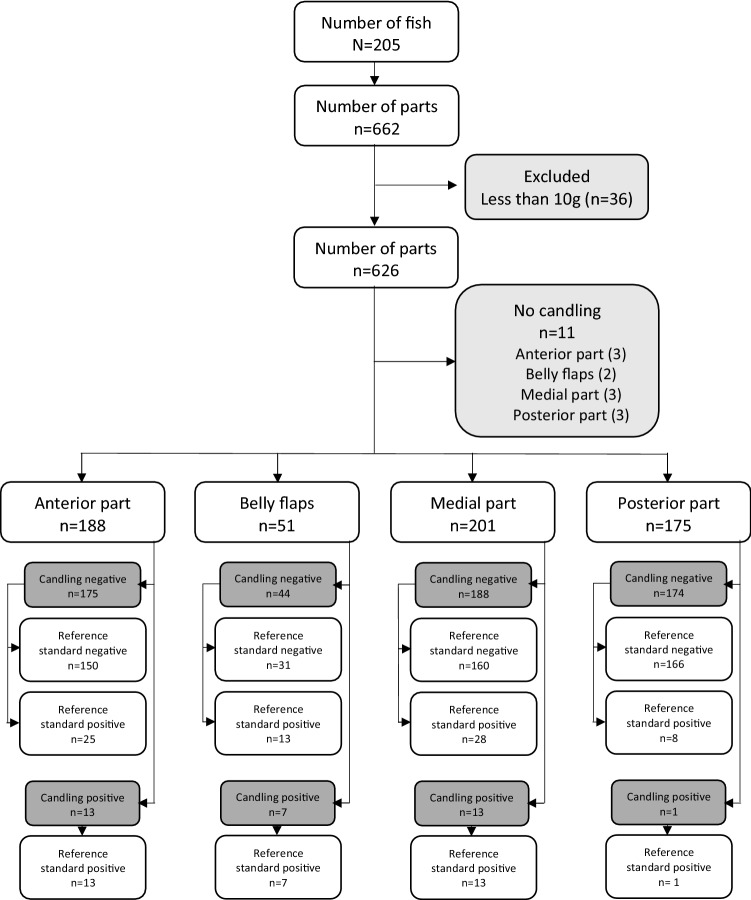
STARD flowchart.

All fish were transported to the laboratory, kept under refrigerated conditions at 4 °C, and processed within 3 to 5 days. Fish were gutted (if this was not already done), and skinned. All fish were divided into an anterior part, belly flap, medial part, and posterior part (left and right side of the fish combined), resulting in 662 fish parts (Fig. 1). Especially for smaller fish, the belly flaps were often merged with the medial part due to the small sizes of these parts.

All fish parts were weighted to the nearest 0.1 g. Fish parts weighing less than 10 g were excluded from the analyses (n = 36) (Fig. 1). On 11 parts, no candling was performed, and these parts were therefore excluded for further analysis.

### Ascaridoid larvae collection

**Figure 2:**
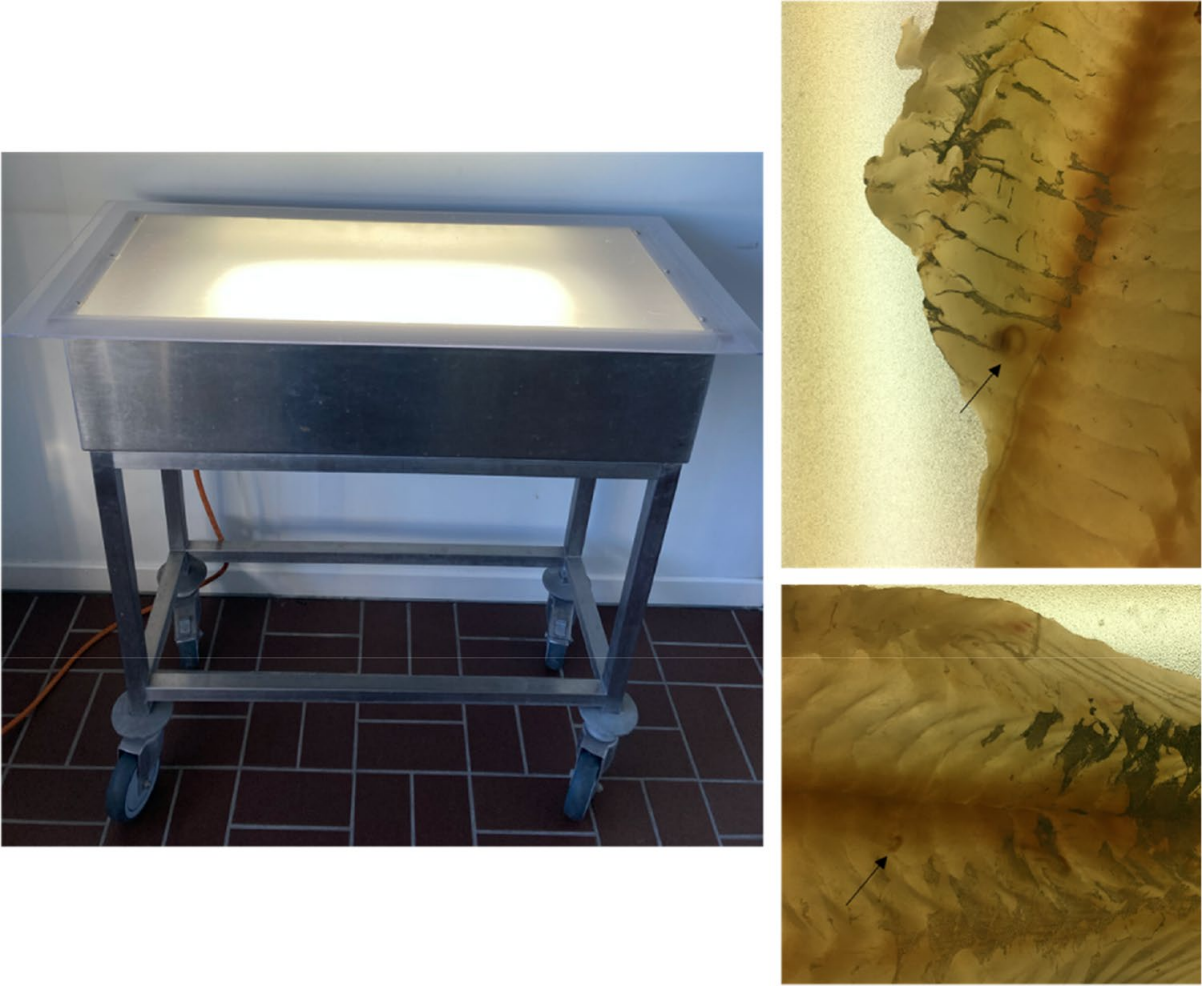
The candling table (left) and examples (right) of visualised embedded ascaridoid larvae in fish muscles (arrow) placed on the candling table.

Candling was performed on 615 fish parts in the laboratory (Fig. 1), under similar conditions as in industrial settings (Fig. 2). The skinless fish parts were placed on a translucent table, illuminated from below with three “cool white” fluorescent lights of 18 W^21,22^. The fish parts were then visually inspected, and larvae were seen as darker than the surrounding fish muscle. Visible larvae were counted and collected. Thereafter, the fish parts were pre-heated for 60–90 min at 44 °C to facilitate digestion. For the digestion, a pepsin/HCl solution (pH 2) was used. Samples were weighted and 2 L of solution was added per 100 g sample^15,20^. After 15 min on a magnetic stirrer at 44 °C, the solution was poured through a sieve and transferred in a petri dish on a light table to count and collect the larvae. Candling and enzymatic digestion was performed by trained researchers (E.M., B.Š., S.V., A.S., T.DS., and J.L).

### Ascaridoid larvae identification

All larvae were identified to the genus level under a light microscope, according to the diagnostic keys of^23,24^. Larvae were preserved in 70% ethanol until further analysis. Of all fish parts, one larva of each morphological identified group (number of larvae, n_l_ = 136) was selected for molecular identification using PCR–RFLP (Polymerase Chain Reaction–Restricted Fragment Length Polymorphism) of the internal transcribed spacer (ITS) fragment as described previously^8^, based on the protocol of the European Reference Laboratory for Parasites^25^. In case of unclear molecular results, sequencing of the ITS fragment was conducted.

### Statistical analysis

Candling and enzymatic digestion were performed separately. A sample was considered positive if at least one larva was detected using candling and/or digestion. Since the infections detected with candling would also be detected with enzymatic digestion, the combination of results from both methods was used as reference standard. The positivity rate was defined as the ratio of the number of infected fish parts to the total number of fish parts and was calculated with 95% confidence interval (Clopper-Pearson) using the DescTools package^26^. For the recovery rate, the total number of larvae detected with both methods were compared with the number of larvae detected solely with candling. Moreover, the sensitivity (Se) and negative predictive value (NPV) were estimated using the epiR package^27^. This for the different fish parts, larval genera, and fish species. Furthermore, we defined the median number of larvae per 100 g infected fish part, with minimum–maximum range (min–max). Logistic regressions were performed to explore the sensitivity of candling for different infection levels, by including the number of larvae/100 g infected muscle as predictor variable, the candling result as outcome, and restricting the analysis to the positive samples (reference standard). The Pearson coefficient was calculated to evaluate the correlation between the number of larvae found in total and those with candling. All significance levels were set at 5% and analyses were conducted in RStudio, using R version 4.0.3^28^.

## Results

### General

The sampling set consisted of 188 anterior parts, 51 belly flaps, 201 medial parts (including the belly flaps of smaller fish still attached), and 175 posterior parts, giving a total of 615 fish parts (Fig. 1). The weight of the fish parts ranged from 10 to 1112 g, with a mean value of 109 g.

The overall positivity rate of the fish parts was 18% (95% CI 15–21%) and the positivity rate with candling as sole detection method was 6% (95% CI 4–8%). The sensitivity of the candling method was 31% (95% CI 23–41%) and the negative predictive value 87% (95% CI 84–90%) (Table 1). No false positive results were observed. The full contingency table can be found in Suppl. Table S1.

**Table 1:** Evaluation of the candling method in the recovery of the different ascaridoid genera in the fish parts (n = 615).

Larvae genus	Infected parts		Positivity rate (%) (95% CI)		Se (%) (95% CI)	n_l_		%C
	C	T	C	T		C	T	
*Anisakis* spp.	19	65	3 (2–5)	11 (8–13)	29 (19–42)	128	581	22
*Pseudoterranova* spp.	11	27	2 (1–3)	4 (3–6)	41 (22–61)	16	42	38
*Hysterothylacium* spp.	1	5	0.2 (0–1)	1 (0.3–2)	20 (0–72)	10	53	19
Mixed infections*	3	11	0.5 (0.1–1)	2 (1–3)	27 (6–61)	20	70	29
Total	34	108	6 (4–8)	18 (15–21)	31 (23–41)	174	746	23

With the number of infected fish parts by candling (C) and total (T); the positivity rate (%) with 95% confidence interval (95% CI); the sensitivity (Se) with 95% confidence interval (95% CI) of candling with the total number of infected parts as reference standard; the number of larvae recovered (n_l_); and the percentage of the total number of larvae that were recovered with candling (%C).

*Mixed infection of *Pseudoterranova* spp., *Anisakis* spp., and *Hysterothylacium* spp. (1); *Pseudoterranova* spp. and Anisakis spp. (9); and *Anisakis* spp. and *Hysterothylacium* spp (1).

**Figure 3:**
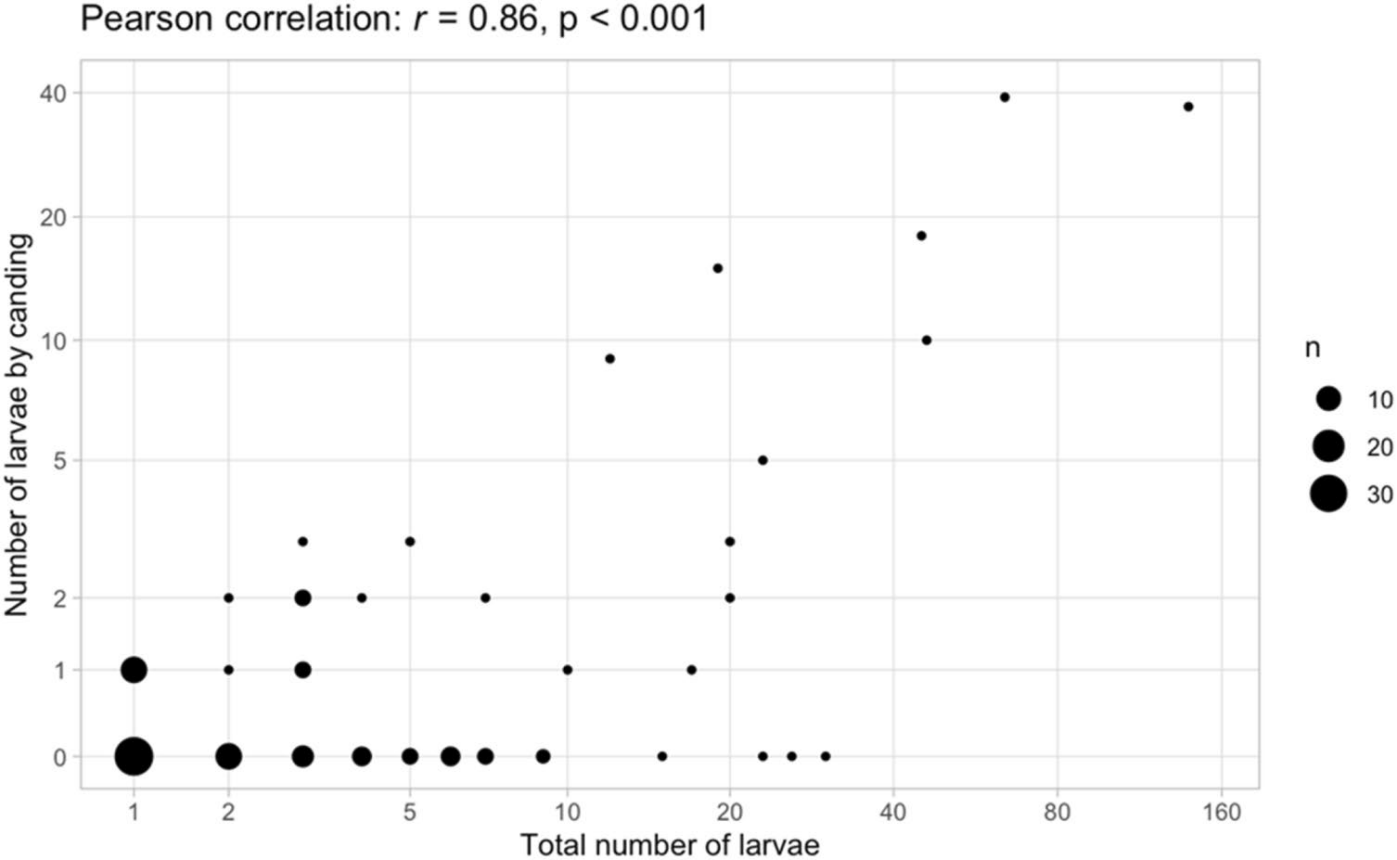
Correlation between the number of ascaridoid larvae detected with candling and the total number, for all infected fish parts (n = sample size), with given Pearson correlation (r) and p-value.

A total of 746 larvae were recovered from the infected fish parts, of which only 174 were detected with candling (23%). A positive correlation between the number of larvae detected in the fish part with candling and the total number of larvae is demonstrated in Fig. 3 (Pearson correlation: *r* = 0.86, p < 0.001).

### Candling performance for the different ascaridoid larvae genera

A subset of 136 larvae (per fish part, one larva of each morphologically identified group) were molecularly identified. Ascaridoid genera recovered were *Anisakis* spp. (*A*. *simplex s.s.* (n_l_ = 80), *A*. *pegreffii* (n_l_ = 1), and *A. hybrid complex* (n_l_ = 4)), *Pseudoterranova* spp. (*P. decipiens s.s*. (n_l_ = 45)), and *Hysterothylacium* spp. (*H. aduncum* (n_l_ = 6)).

The highest sensitivity of candling was observed for *Pseudoterranova* spp. larvae (41% (95% CI 22–61%), Table 1). The point estimate for *Anisakis* spp. seemed lower, with 29% (95% CI 19–42%) (Table 1). Regarding the number of larvae recovered via candling, likewise this was the highest for *Pseudoterranova* spp. larvae (38%). The recovery of *Anisakis* spp. and *Hysterothylacium* spp. larvae was similar, with approximately 20% of all larvae retrieved.

### Candling performance on the different fish parts

**Figure 4:**
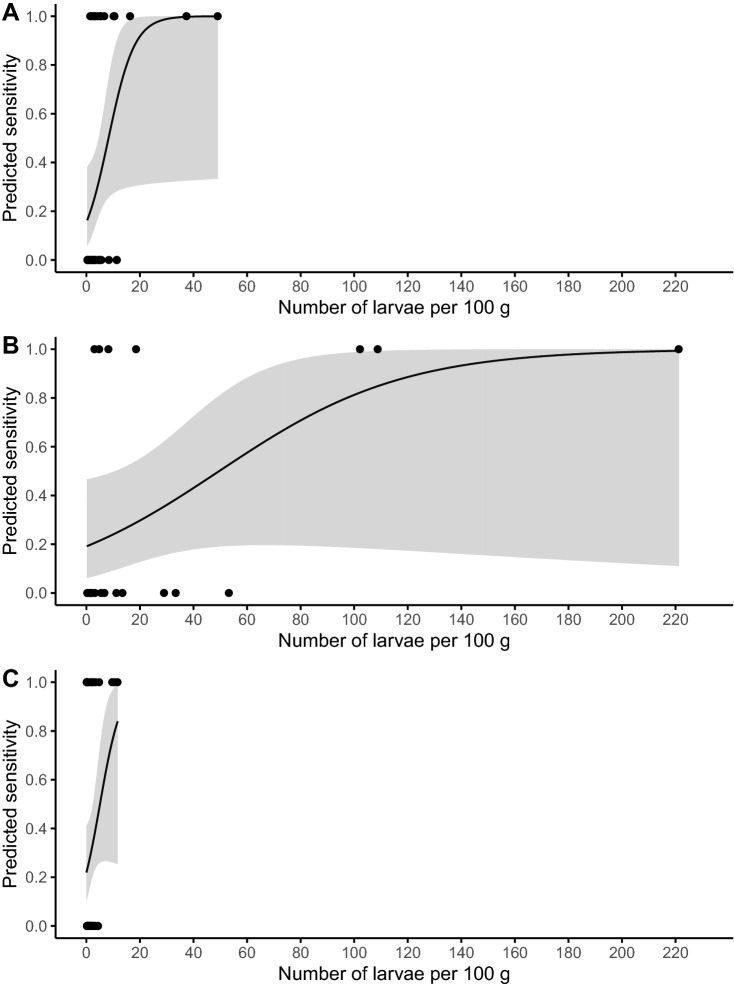
Predicted sensitivity and 95% confidence interval (grey zone) of candling for the number of ascaridoid larvae in 100 g infected muscle. With (**A**) Anterior part; (**B**) Belly flaps; and (**C**) Medial part.

The sensitivity of candling was calculated for the different fish parts. Similar sensitivities were determined for the belly flaps (35%), anterior part (34%), and medial part (32%) (Table 2). The median number of larvae/100 g infected muscle was seven larvae (min–max: 0.3–221) in the belly flaps, three larvae (0.2–49) in the anterior part, and two larvae in the medial (0.1–12) and posterior part (0.3–25). In Fig. 4, the predicted Se is plotted for the number of larvae/100 g infected sample. An increase in Se for higher numbers of larvae/100 g infected part is observed for all fish parts. The posterior part is not shown in Fig. 4 due to the limited number of positive samples. In the belly flaps, despite having a higher number of larvae/100 g in infected parts than the other fish parts, the overall Se was similar (Table 2) and some highly infected parts (n_i_ = 3, with numbers varying between 29 and 53 larvae/100 g) were not detected with candling (Fig. 4). The highest NPV was observed in the posterior part (95% (95% CI 91–98)), where also the lowest positivity rate was found. The highest proportion of larvae recovered by candling was in the medial part (29%), and the least in the posterior part (13%) (Table 2).

**Table 2:** Evaluation of the candling method in the recovery of ascaridoid larvae in the different fish parts.

Fish part	n	Infected parts		Positivity rate (%) (95% CI)		NPV (%) (95% CI)	Se (%) (95% CI)	n_l_		% C
		C	T	C	T			C	T	
Anterior part	188	13	38	7 (4–12)	20 (15–27)	86 (80–91)	34 (20–51)	48	250	19
Belly flaps	51	7	20	14 (6–26)	39 (26–54)	70 (55–83)	35 (15–59)	70	297	24
Medial part	201	13	41	6 (3–11)	20 (15–27)	85 (79–90)	32 (18–48)	54	184	29
Posterior part	175	1	9	1 (0.01–3)	5 (2–10)	95 (91–98)	11 (0–48)	2	15	13
Total	615	34	108	6 (4–8)	18 (15–21)	87 (84–90)	31 (23–41)	174	746	23

With the number of fish parts (n); the number of infected fish parts by candling (C) and total (T); the positivity rate (%) with 95% confidence interval (95% CI); the negative predictive value (NPV) and sensitivity (Se) of candling with 95% confidence interval; the number of larvae (n_l_); and the percentage of the total number of larvae that were recovered with candling (%C).

**Figure 5:**
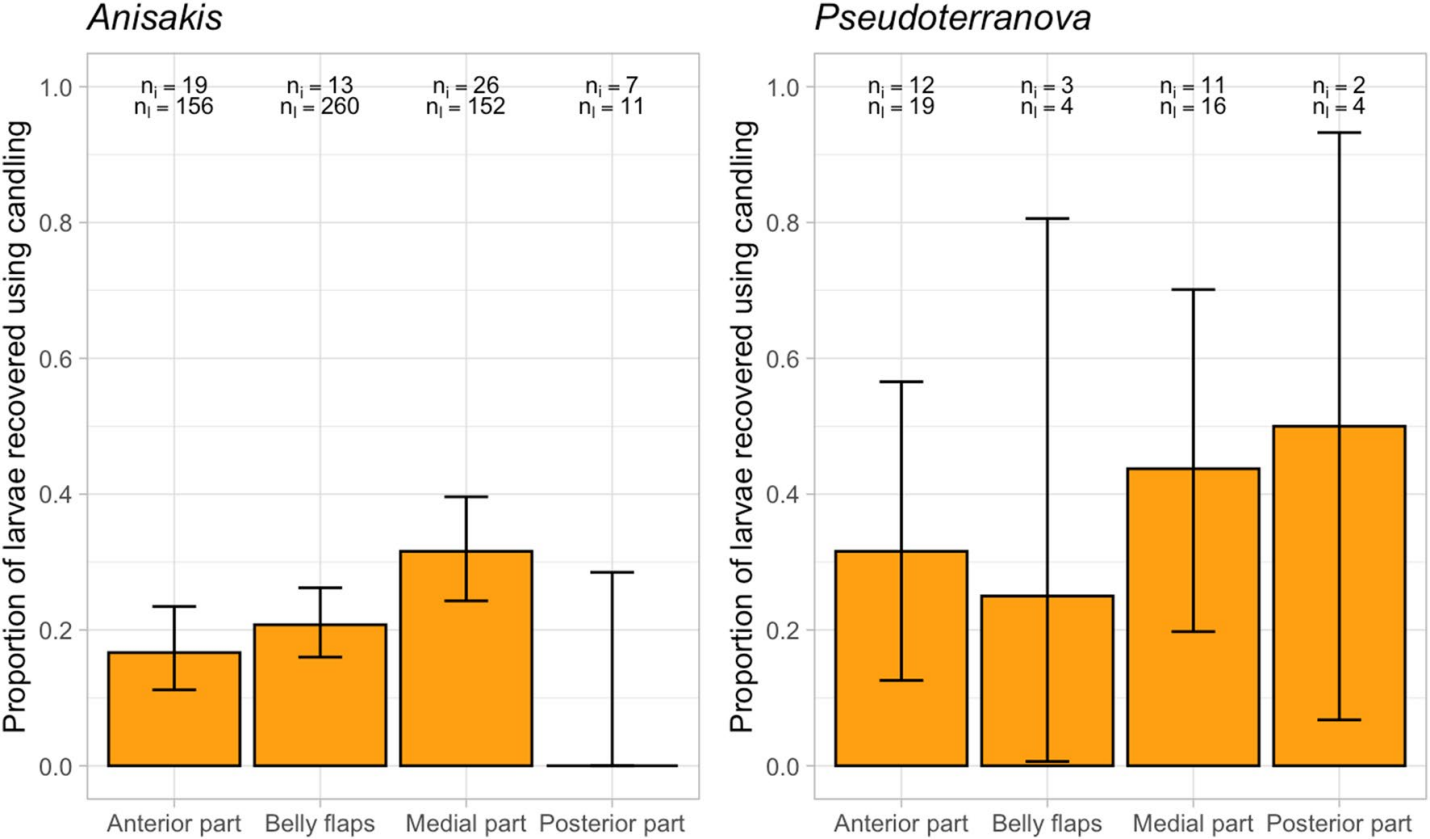
Proportion of ascaridoids recovered by candling on genus level for each fish part, including mixed (*Anisakis* spp. and *Pseudoterranova* spp.) infections. With *n*_*i*_ number of infected parts; *n*_*l*_ total number of larvae for each infected part.

We further evaluated the performance of the candling method on the different larval genera in the different fish parts. We found that the Se of the detection of *Anisakis* spp. was slightly higher in the belly flaps, compared to the medial part (Table S2). For other fish parts and larval species, the number of positive samples was too low to draw solid conclusions (Table S2). In all parts, larvae recovery of the *Pseudoterranova* spp. seemed higher than the recovery of the *Anisakis* spp. (Fig. 5).

### Candling performance for the different fish species

To explore potential fish species effects, we analysed the Se and NPV for the different fish species. Fish parts were collected from 32 commercial fish species of which 13 were found to be ascaridoid-free (Table S3). Despite the low sample sizes, we highlight the candling performance of a few species with adequate number (> 10) of positive fish parts (Table S3). In gurnard (*Chelidonichthys cuculus*) and monkfish (*Lophius piscatorius*) fish parts, a Se of respectively 36% (95% CI18-57) and 31% (95% CI9-61) was found, and the number of larvae was underestimated (28 and 21% of the total larvae found). In pollack (*Pollachius pollachius*), the NPV and Se were 57% (95% CI 37–75) and 32% (95% CI 13–57%), respectively.

## Discussion

Today, candling is the standard method for the detection and removal of ascaridoid larvae on an industrial scale. Our results show a sensitivity of 31% (95% CI 23–41%) and a recovery of 23% of the larvae. Comparison of candling with other detection methods in the past showed a low efficacy as well. Levsen et al.^16^ compared the candling method with enzymatic digestion and UV-press method in herring (*Clupea harengus*), mackerel (*Scomber scombrus*) and blue whiting (*Micromesistius poutassou*) and found that only 7–10% of the *Anisakis simplex* larvae were recovered with candling. We examined herring and mackerel as well, with respectively one out of two larvae and one out of 19 larvae recovered. For both fish species, the number of infected fish parts and the number of larvae recovered was low, hampering proper comparison. As defining the candling performance in different fish species was not our primary focus, obtaining a sufficient sample size for each fish species was not envisaged. Petrie et al.^17^ on the other hand, recovered 50% of the larvae with candling compared with slicing for monkfish and cod. Slicing is a detection method that overcomes the candling limitation of the thickness of the fish fillet, by slicing it transversely into 0.5–1 cm thin fillets. Reported larvae recovery with this method is 78–84%, in comparison with additional enzymatic digestion to recover all larvae^29,30^. Therefore, the candling results by Petrie et al.^17^ may be overestimated. In conclusion of the study of Petrie et al.^16^, candling gave better results when candling the belly flaps of monkfish, while candling the belly flaps of herring and mackerel was described as impractical due to, among other things, the dark colour of the fish. The latter was also identified as a limiting factor by Levsen et al.^16^ besides texture and/or the location of the larvae.

To evaluate the effect of the location of the larvae, we divided our samples in an anterior, belly flaps, medial, and posterior part. The Se and larvae recovery were lowest for the posterior part, but this location was barely infected, probably due to the long distance from the viscera, and therefore the Se comes with a higher uncertainty. Nevertheless, candling of this part could be advised against since it may not be cost-effective to detect the few infections. The belly flaps were the highest infected region, with primarily *Anisakis* spp. Given the denser structure of this part, larvae are mainly visible when lodged on the upper or outer surface (body cavity) of the belly flaps. The lesser visibility of embedded larvae was also demonstrated by the fact that theoretically more than 220 larvae are needed to reach a Se of one. Moreover, highly infected belly flaps were not always detected with candling (a fish part with 53 larvae/100 g muscle was not detected). A low NPV was found in this part, therefore a negative candling result should still be considered as potentially infected. Some authors have even suggested to remove the belly flaps before human consumption^31–33^. Based on our results, given the generally higher levels of infection with primarily *Anisakis* spp. (i.e. the species most linked with pathogenicity), the very low ability to pick up infection, and an insufficient larval recovery in the belly flaps with candling, we believe candling to be insufficiently efficacious in this part and advise freezing or indeed removal before consumption.

The positivity rate as well as the Se was similar for the anterior and medial part. Since these parts make up the majority of the fish fillet, proper candling remains important to reduce the number of infected fillets entering the food chain by a third and the number of larvae by a fourth.

In addition to the fish host and the fish part, other factors possibly influencing candling have been investigated such as the thickness of the fish fillet. Transmitted candling light decreases with thicker fillets^34^ and the detection limit for embedded larvae is estimated around 0.4–0.6 cm depth by laboratory experiments^35,36^, whereas the European Food Safety Authority (EFSA) described candling effective up to 2.5 cm^1^. White transmitted light is still the most advantageous, despite limited contrast between the larvae and the fish muscles due to scattering of the light^34,35,37^. Remark that in this study, the size and thickness of the fillet was fish host dependent.

As final comparison, our own study of candling on cod (*Gadus morhua*) belly flaps showed a Se of 34% (26–42%) at retail level^38^. On larvae species level, both the Se and number of larvae recovered were similar for *Anisakis* spp. and *Pseudoterranova* spp. In that study, fish were candled prior to our acquisition, therefore an underestimation of the *Pseudoterranova* spp. was possible^38^. In the current study, the recovery of *Pseudoterranova* spp. larvae was the most effective, both in terms of presence of infection as number of larvae. The higher detection of the genus *Pseudoterranova* was expected since they are generally reddish to brown and thicker than *Anisakis* spp. (whitish and often coiled-up)^39,40^. Removal of *Pseudoterranova* spp. larvae does not only reduce the health risk but also has an impact on the aesthetic concerns of the consumers, in comparison with the less easy to spot *Anisakis* spp. larvae. Still, the proportion of *Pseudoterranova* spp. was low (11%) with an average of two larvae/100 g infected muscle. On fish species level, the proportion of *Pseudoterranova* spp. larvae was high in gurnard and cod. Candling is advised in these fish species since the larvae should be easier to spot.

Only 23% of the total number of larvae were recovered with candling. Based on the positive correlation between the number of larvae found with candling and in total, candling could be a predictor of the intensity of infection though the overall reliability could be questioned. Llarena-Reino et al. investigated whether or not the number of larvae in the viscera can be predictive for the larvae in the muscles, but also there no statistical significance was found^41^.

Given the limitations of candling, especially in the detection of low intensity infections, the process of larvae detection should be revised. Rough set theory has been applied, assuming a relation between the presence of infection and the fitness of the fish, whereby patterns between larvae species and catch location and/or water salinity were obtained^42^. This work is still in his infancy and a database of infection numbers and parameters is needed for multiple fish species to ‘teach’ the artificial intelligence system. Other risk-based systems have also been developed aiming for a more targeted use of candling or of freezing the fish^43^. A first risk categorization scheme has been developed by Llarena-Reino et al.^18^ calling it the SADE (Site of infection, the Assurance of quality (pathological and commercial), Density of infection, and Epidemiological relevance of the fish species) scoring system. Fish batches are scored, with score ten meaning parasite-free lots and score zero indicating the need to reprocess the fish to assure food safety. The main goal of this flow chart is to standardise the inspection in the fish industry. A second scoring system, Fish Parasite Rating (FPR) has been developed by Rodríguez et al.^44^, categorizing fish lots into five groups. Data is obtained from visual inspection and enzymatic digestion, with results showing only in four lots (of 19) a rejection not observed with visual inspection. In our study, we observed the influence of the fish host, larvae species, and site of infection, though did not apply the above scoring systems since our objective was to analyse the candling method on all available fish species/parts.

This study investigated the performance of candling. We assessed whether the current candling method could be used in a more targeted way, focusing on fish parts and fish/larvae species where candling would have the highest efficacy. We advise thorough candling of the anterior and medial part, and the removal or freezing of the belly flaps. Candling had a higher efficacy in the detection of *Pseudoterranova* spp. but these larvae species were underrepresented in comparison with the harder to spot *Anisakis* spp.

## Conclusion

In conclusion, we determined an overall Se of 31% (95% CI 23–41%) and a NPV of 87% (95% CI 85–90%) for the candling technique. The lowest NPV was estimated for the detection of Anisakidae in the belly flaps and the highest Se for the detection of *Pseudoterranova* spp. larvae. European regulation (EC) No 853/2004 states the removal of all visible larvae and the discarding of heavily infected fish^14^. Though this regulation does not provide more details on these criteria, our results show that candling can be used to detect the more heavily infected fish, so it could be a valuable screening method to remove these fish from the market. Nevertheless, due to the low sensitivity for lowly infected fish parts, emphasis is placed on the need to either heat or freeze the fish fillets to adequate temperature since candling has its shortcomings. An in-depth assessment of the cost benefits and consumers’ health risk reduction of candling for the different fish parts and fish species needs to be further conducted.

## Supplementary Information

Supplementary Tables.
