# Supplementary material for: Sensitivity of candling as routine method for the detection and recovery of ascaridoids in commercial fish fillets
Source: Sci Rep. 2022 Jan 25;12:1358. doi: 10.1038/s41598-022-05235-6 (PMC8789850; doi:10.1038/s41598-022-05235-6)
Supplement: Supplementary file 1 — Supplementary Tables. [file 41598_2022_5235_MOESM1_ESM.docx]

**Supplementary tables**

Table S1: Contingency table of fish muscle infections with ascaridoid larvae

A: All fish parts; B: *Anisakis* spp.; C: *Pseudoterranova* spp.; D: *Hysterothylacium* spp.; E: mixed infections (*Pseudoterranova* spp., *Anisakis* spp., and *Hysterothylacium* spp. (1); *Pseudoterranova* spp. and *Anisakis* spp. (9); and *Anisakis* spp. and *Hysterothylacium* spp. (1)); F: Anterior part; G: Medial part; H: Posterior part; I: Belly flaps.

|  |  | **Candling** | | |  |  | **Candling** | | |
| --- | --- | --- | --- | --- | --- | --- | --- | --- | --- |
|  | **A** | Positive | Negative | Total |  | **B** | Positive | Negative | Total |
| **Total** | Positive | 34 | 74 | 108 | **Total** | Positive | 19 | 46 | 65 |
|  | Negative | 0 | 507 | 507 |  | Negative | 0 | 550 | 550 |
|  | Total | 34 | 581 | 615 |  | Total | 19 | 596 | 615 |
|  |  | **Candling** | | |  |  | **Candling** | | |
|  | **C** | Positive | Negative | Total |  | **D** | Positive | Negative | Total |
| **Total** | Positive | 11 | 16 | 27 | **Total** | Positive | 1 | 4 | 5 |
|  | Negative | 0 | 588 | 588 |  | Negative | 0 | 610 | 610 |
|  | Total | 11 | 604 | 615 |  | Total | 1 | 614 | 615 |
|  |  | **Candling** | | |  |  | **Candling** | | |
|  | **E** | Positive | Negative | Total |  | **F** | Positive | Negative | Total |
| **Total** | Positive | 3 | 8 | 11 | **Total** | Positive | 13 | 25 | 38 |
|  | Negative | 0 | 604 | 604 |  | Negative | 0 | 150 | 150 |
|  | Total | 3 | 612 | 615 |  | Total | 13 | 175 | 188 |
|  |  | **Candling** | | |  |  | **Candling** | | |
|  | **G** | Positive | Negative | Total |  | **H** | Positive | Negative | Total |
| **Total** | Positive | 13 | 28 | 41 | **Total** | Positive | 1 | 8 | 9 |
|  | Negative | 0 | 160 | 160 |  | Negative | 0 | 166 | 166 |
|  | Total | 13 | 188 | 201 |  | Total | 1 | 174 | 175 |
|  |  | **Candling** | | |  |  |  |  |  |
|  | **I** | Positive | Negative | Total |  |  |  |  |  |
| **Total** | Positive | 7 | 13 | 20 |  |  |  |  |  |
|  | Negative | 0 | 31 | 31 |  |  |  |  |  |
|  | Total | 7 | 44 | 51 |  |  |  |  |  |

Table S2 – Evaluation of the candling method in the recovery of the different ascaridoid genera for each fish part.

With the number of positive fish parts detected by candling (C) and in total (T); The sensitivity (Se) of candling with 95% confidence interval [95%-CI]. With NA – not applicable and *Mixed infection of *Pseudoterranova* spp., *Anisakis* spp., and *Hysterothylacium* spp. (1); *Pseudoterranova* spp. and *Anisakis* spp. (9); and *Anisakis* spp. and *Hysterothylacium* spp. (1).

| **Larval genus** | **Anterior part (n = 188)** | | | **Belly flaps (n = 51)** | | | **Medial part (n = 201)** | | | **Posterior part (n=175)** | | |
| --- | --- | --- | --- | --- | --- | --- | --- | --- | --- | --- | --- | --- |
|  | **C** | **T** | **Se** | **C** | **T** | **Se** | **C** | **T** | **Se** | **C** | **T** | **Se** |
| *Anisakis* spp. | 6 | 19 | 32 [13-57] | 5 | 13 | 38 [14-68] | 8 | 26 | 31 [14-52] | 0 | 7 | 0 [0-41] |
| *Pseudoterranova* spp. | 4 | 12 | 33 [10-65] | 1 | 3 | 33 [1-91] | 5 | 10 | 50 [19-81] | 1 | 2 | 50 [1-99] |
| *Hysterothylacium* spp. | 1 | 2 | 50 [1-99] | 0 | 1 | 0 [0-97] | 0 | 2 | 0 [0-84] | 0 | 0 | NA |
| Mixed infections* | 2 | 5 | 40 [5-85] | 1 | 3 | 33 [1-91] | 0 | 3 | 0 [0-71] | 0 | 0 | NA |

Table S3 – Evaluation of the candling method for the detection of ascaridoid infection and intensity of infection in fish muscle samples.

With the number of fish parts (n); the number of infected fish parts by candling (C) and total (T); the positivity rate with 95% confidence interval [95%-CI]; the negative predictive value (NPV) and sensitivity (Se) of candling with 95% confidence interval; the median number of larvae per 100g infected fish part with minimum-maximum range [min-max]; the percentage of larvae found with candling (%C); and the total number of larvae (n_l_) for each larvae genus, with A. spp.-*Anisakis* spp.; P. spp.-*Pseudoterranova* spp.; and H. spp.-*Hysterothylacium* spp.

| **Fish species** | **n** | **Infected** | | **Positivity rate (%) [95%-CI]** | | **NPV (%)  [95%-CI]** | **Se (%)   [95%-CI]** | **Larvae/100g [min-max]** | | **% C** | **n_l_** | | |
| --- | --- | --- | --- | --- | --- | --- | --- | --- | --- | --- | --- | --- | --- |
|  |  | **C** | **T** | **C** | **T** |  |  | **C** | **T** |  | **A. spp.** | **P. spp.** | **H. spp.** |
| Sole (*Solea solea)* | 56 | 0 | 0 | 0 [0-6] | 0 [0-6] | - | - | - | - | - | - | - | - |
| Gurnard (*Triglidae* spp.) | 59 | 9 | 25 | 15 [7-27] | 42 [30-56] | 68 [53-80] | 36 [18-57] | 3.3 [1.4-221.2] | 4.4 [0.6-269.2] | 28 | 37 | 41 | 51 |
| Plaice (*Pleuronectes platessa)* | 38 | 0 | 2 | 0 [0-9] | 5 [1-18] | 95 [82-99] | 0 [0-84] | - | 13.6 [1.8-25.4] | 0 | 5 | 1 | - |
| Herring (*Clupea harengus)* | 35 | 1 | 2 | 3 [0.1-15] | 6 [1-19] | 97 [85-100] | 50 [1-99] | 2.3 [2.3-2.3] | 5.1 [4.6-5.5] | 50 | 2 | - | - |
| Lemon sole (*Microstomus kitt)* | 32 | 0 | 1 | 0 [0-11] | 3 [0.1-16] | 97 [84-100] | 0 [0-97] | - | 1.7 [1.7-1.7] | 0 | - | 1 | - |
| Seabass (*Dicentrarchus labrax)* | 37 | 0 | 1 | 0 [0-9] | 3 [0.1-14] | 97 [86-100] | 0 [0-97] | - | 2 [2-2] | 0 | 1 | - | - |
| Goldbrasse (*Sparus aurata)* | 34 | 0 | 0 | 0 [0-10] | 0 [0-10] | - | - | - | - | - | - | - | - |
| Mackerel (*Scomber scombrus)* | 30 | 1 | 7 | 3 [0.1-17] | 23 [9-42] | 79 [60-92] | 14 [0-58] | 2.0 [2.0-2.0] | 2.7 [1.4-33.3] | 5 | 14 | - | 5 |
| Monkfish (*Lophius piscatorius)* | 34 | 5 | 13 | 12 [3-27] | 38 [22-56] | 70 [51-85] | 31 [9-61] | 0.4 [0.2-108.8] | 1.1 [0.2-137.7] | 21 | 193 | 7 | - |
| Pollack (*Pollachius Pollachius)* | 36 | 6 | 19 | 17 [6-33] | 53 [35-70] | 57 [37-75] | 32 [13-57] | 9.3 [3.0-18.5] | 2.1 [0.2-53.2] | 33 | 204 | 13 | - |
| Turbot (*Psetta maxima)* | 20 | 0 | 0 | 0 [0-17] | 0 [0-17] | - | - | - | - | - | - | - | - |
| Brill (*Scophthalmus rhombus)* | 18 | 0 | 0 | 0 [0-19] | 0 [0-19] | - | - | - | - | - | - | - | - |
| Saithe (*Pollachius virens)* | 21 | 1 | 7 | 5 [0.1-24] | 33 [15-57] | 70 [46-88] | 14 [0-58] | 0.1 [0.1-0.1] | 0.5 [0.2-13.5] | 3 | 38 | - | - |
| Sardine (*Sardina pilchardus)* | 6 | 0 | 0 | 0 [0-46] | 0 [0-46] | - | - | - | - | - | - | - | - |
| Witch flounder (*Glyptocephalus cynoglossus)* | 17 | 0 | 1 | 0 [0-20] | 6 [0.1-29] | 94 [71-100] | 0 [0-97] | - | 0.9 [0.9-0.9] | 0 | 1 | - | - |
| Dogfish (*Scyliorhinus* spp.) | 17 | 0 | 0 | 0 [0-20] | 0 [0-20] | - | - | - | - | - | - | - | - |
| Halibut (*Hippoglossus hippoglossus)* | 16 | 2 | 8 | 12 [16-38] | 50 [25-75] | 57 [29-82] | 25 [3-65] | 2.8 [0.4-5.2] | 0.7 [0.2-11.3] | 8 | 63 | - | - |
| Pouting (*Trisopterus luscus)* | 15 | 1 | 3 | 7 [0.2-32] | 20 [4-48] | 86 [57-98] | 33 [1-99] | 4.1 [4.1-4.1] | 5.1 [0.9-8.2] | 12 | 7 | 2 | - |
| Common dab (*Limanda limanda)* | 8 | 2 | 2 | 25 [3-65] | 25 [3-65] | 100 [54-100] | 100 [16-100] | 10.9 [10.2-11.6] | 18.2 [16.9-19.4] | 67 | 1 | 5 | - |
| Rainbowtrout (*Oncorhynchus mykiss)* | 13 | 0 | 0 | 0 [0-25] | 0 [0-25] | - | - | - | - | - | - | - | - |
| Sea trout (*Salmo trutta trutta)* | 12 | 0 | 0 | 0 [0-26] | 0 [0-26] | - | - | - | - | - | - | - | - |
| Greater weever (*Trachinus* spp.) | 9 | 3 | 3 | 33 [7-70] | 33 [7-70] | 100 [54-100] | 100 [29-100] | 16.3 [9.6-49.0] | 21.7 [12.9-53.9] | 19 | 16 | - | - |
| Redfish (*Sebastes spp)* | 10 | 2 | 5 | 20 [3-56] | 50 [19-81] | 62 [24-91] | 40 [5-85] | 5.0 [4.7-5.3] | 3.3 [2.2-10.7] | 29 | 14 | 3 | - |
| (European) Flounder (*Platichthys flesus)* | 5 | 0 | 0 | 0 [0-52] | 0 [0-52] | - | - | - | - | - | - | - | - |
| Arctic char (*Salvelinus alpinus)* | 6 | 0 | 0 | 0 [0-46] | 0 [0-46] | - | - | - | - | - | - | - | - |
| Atlantic salmon (*Salmo salar)* | 6 | 0 | 0 | 0 [0-46] | 0 [0-46] | - | - | - | - | - | - | - | - |
| Cod (*Gadus morhua)* | 6 | 2 | 4 | 33 [4-78] | 67 [22-96] | 50 [7-93] | 50 [7-93] |  | 0.6 [0.6-2.9] | 30 | 1 | 9 | - |
| Garfish (*Belone belone)* | 6 | 0 | 2 | 0 [0-46] | 33 [4-78] | 67 [22-96] | 0 [0-84] | - | 2.4 [1.7-3.2] | 0 | 6 | - | - |
| Haddock (*Melanogrammus aeglefinus)* | 4 | 0 | 0 | 0 [0-60] | 0 [0-60] | - | - | - | - | - | - | - | - |
| Horse mackerel (*Trachurus* spp.) | 3 | 0 | 1 | 0 [0-71] | 33 [1-91] | 67 [9-99] | 0 [0-97] | - | 7.4 [7.4-7.4] | 0 | 1 | - | - |
| Leng (*Molva molva)* | 3 | 0 | 2 | 0 [0-71] | 67 [9-99] | 33 [1-99] | 0 [0-84] | - | 0.5 [0.4-0.6] | 0 | 4 | - | - |
| Whiting (*Merlangius merlangus)* | 3 | 0 | 0 | 0 [0-71] | 0 [0-71] | - | - | - | - | - | - | - | - |
| **Total** | **398** | **35** | **109** | **6 [4-8]** | **18 [15-21]** | **87 [84-90]** | **31 [23-41]** | **4.7 [0.1-221.2]** | **2.7 [0.2-269.2]** | **23** | **608** | **82** | **56** |
